# The Momentum trial: the efficacy of using a smartphone application to promote patient activation and support shared decision making in people with a diagnosis of schizophrenia in outpatient treatment settings: a randomized controlled single-blind trial

**DOI:** 10.1186/s12888-019-2143-2

**Published:** 2019-06-17

**Authors:** Tobias Vitger, Stephen F. Austin, Lone Petersen, Esben S. Tønder, Merete Nordentoft, Lisa Korsbek

**Affiliations:** 1 Competence Centre for Rehabilitation and Recovery, The Mental Health Centre Ballerup, The Mental Health Services of the Capital Region, Ballerup, Denmark; 2 Psychiatric Research Unit, Region Zealand Psychiatry, Slagelse, Denmark; 3 The Mental Health Centre Slagelse, The Mental Health Services of Zealand, Slagelse, Denmark; 4 The Research Unit of the Mental Health Centre Copenhagen, The Mental Health Services of the Capital Region, Copenhagen, Denmark

**Keywords:** Shared decision making, Patient activation, Schizophrenia, Schizotypal, Early intervention, First-episode psychosis, mHealth, Randomized clinical trial

## Abstract

**Background:**

Shared decision making (SDM) is often defined as an interactive process that ensures that both patient and practitioner are actively involved in the treatment and that they share all relevant information to arrive at a mental health decision. Previous SDM interventions have found improvements in outcomes such as personal recovery, higher perceived involvement in treatment decisions and knowledge about one’s disease. Still, SDM occurs less frequently in mental health care than in primary care. Electronic aids developed to support patient activation and SDM could be a promising mean to engage patients in their mental healthcare.

The aim of this trial is to investigate the effects of using a smartphone app to promote patient activation and support SDM for people with schizophrenia-spectrum disorders in an outpatient treatment setting.

**Methods:**

This randomised controlled trial will allocate participants to one of two groups: (1) Intervention group: smartphone app and TAU (treatment as usual) or (2) Control group: TAU without the smartphone app. A total sample size of 260 people with a diagnosis of schizophrenia, schizotypal or delusional disorder will be recruited from five OPUS teams (a specialized early intervention program) in Denmark between 2019 and 2020. The intervention will last for 6 months with data collection at baseline, and at 3 and 6 months. Primary outcome will be self-perceived patient activation. Secondary outcomes will be feeling of being prepared for SDM; self-efficacy; working alliance; treatment satisfaction; positive and negative symptoms; level of functioning; hope; and perceived efficacy in patient-provider interaction. Patients’ and health providers’ preferences in clinical decision making will be assessed. Patients’ usage and perceived usefulness of the app will be explored.

**Discussion:**

This study will investigate the efficacy of using the smartphone app to support people with severe mental illness in engaging in their own healthcare management. The study may provide evidence to the idea that linking client and practitioner in digital solutions can have advantages in facilitating SDM in mental health. The trial will provide new knowledge of whether a digital healthcare solution can improve patient activation and support SDM for people with severe mental illness.

**Trial registration:**

ClinicalTrials.gov number: NCT03554655 Registered on: June 13, 2018.

## Background

Shared decision making (SDM) is often defined as an interactive process that ensures that both patient and practitioner are actively involved in the treatment and that they share all relevant information in order to arrive at a mental health decision [1, 2]. While practitioners provide a professional expertise with information on the diagnosis, course of the illness, treatment options and potential side effects, patients are experts on their own needs, treatment preferences and goals [1]. Working together with their unique competences, SDM can be conceptualized as an intermediate position on a continuum with clinician-led decisions at one end and patient-led decisions at the other end [3].

In mental healthcare, SDM has been assessed as a promising treatment intervention with research studies indicating that SDM interventions may promote personal recovery [4], lead to more positive attitude towards medication [5], improve adherence with treatment [5], improve knowledge about the disease [6], lead to a higher perceived involvement in treatment decisions [6, 7], promote patient activation in treatment consultations [8] and may increase satisfaction with treatment decisions [7]. On a more ethical note, becoming more active and engaged in one’s own healthcare is an important element for the patient to (re)gain control and responsibility over his/her own life and recovery process [9]. In addition, clinical guidelines advocate the use of SDM as a patient-centered mental healthcare [10, 11].

Although the current evidence on the efficacy of SDM interventions in mental healthcare appears to be encouraging, the evidence is limited mainly due to a lack of high quality RCT studies and implementation challenges [3, 12]. A central challenge for studies investigating SDM is the complexity of SDM since addressing all of its components into one trial might not be feasible. SDM is a highly complex intervention including a wide range of characteristics (defining the problem, outlining options, reaching a mutual agreement etc.) while also being dependent on a number of mechanics surrounding the patient and provider (e.g. alliance, communication, preferences and engagement) [13]. Research studies often tend to only focus on certain components of SDM (e.g. SDM training for providers or a decision aid for patients) [13], which could help explain why many RCTs are not finding differences between intervention and control subjects.

Although clinical guidelines encourage mental healthcare providers to engage patients in treatment decisions, only few healthcare providers consistently attempt to engage patients in their treatment or adjust the care to the patient’s preferences [14]. This may contribute to the discrepancy between SDM being a preferred model for clinical decision-making for many patients and providers in mental healthcare [15, 16] and patients indicating that they are not involved as much as they want to be in their treatment [17]. The feeling of being involved or as an active participant in one’s mental healthcare may improve affective-cognitive outcomes (e.g. alliance with provider or satisfaction with care), behavioural outcomes (e.g. engagement in treatment such as decision making) and health outcomes (e.g. symptom reduction) [18]. In addition, active or engaged patients with higher expectations for collaborative care could also activate their provider in more collaborative care, thereby creating a good foundation for SDM [19].

One potential mean to facilitate SDM in mental healthcare could be the use of mHealth (mobile healthcare), thereby linking patients and providers together in computer-mediated interventions [20, 21]. A majority of younger psychiatric patients (< 45 years old) indicates an interest in using smartphone apps to evaluate their mental health [22] and a Cochrane review on decision aids (not only mHealth) found improvements in participant’s level of knowledge, patient-clinician communication, participation in treatment and satisfaction with the decision-making process [23].

Few studies have investigated the use of digital solutions in facilitating SDM in mental health and the evidence on the beneficial and possible harmful effects of using such tools in people with severe mental illness are scarce and limited by a lack of randomized controlled trials (RCT). These RCTs do, however, suggest that electronic aids to support SDM are a promising mean to engage patients in their mental health treatment [20, 24–26]. Multiple systematic reviews highlight a need for more evidence-based research on the efficacy and effectiveness of mental health apps [27–30].

This protocol describes a RCT to investigate the effects of using a smartphone app to promote patient activation and support SDM in mental healthcare. Engaged or active patients have been defined as patients being able to; take an active role in the treatment; collaborate with their mental healthcare provider (e.g. communicate concerns, needs and preferences, discuss treatment options and make treatment decisions) and; manage their symptoms and condition [31].

The system being used in the trial consists of a smartphone app (for the patient) and a web portal (for the provider). Information entered in the app by the patient will automatically be transferred to the web portal for the provider to see. This procedure will give the patient and the provider an option for a common starting point when meeting for consultations. Since decisions in mental healthcare often are an ongoing process, the smartphone app is not a classic decision aid focusing on finding a choice to a one-off decision at a single point in time. Instead, the smartphone app presented in this trial is an aid to support some of the underlying elements behind SDM such as engagement in care, collaboration with one’s provider, awareness and eliciting of one’s needs, preferences and values. The app consists of the following functions:

- Questions with multiple choices to prepare for treatment consultation (e.g. what have been important to you since your last visit?)
- Daily self-evaluation on parameters such as stress, sleep and well-being
- Plans for action (e.g. recovery goal setting, personal strategies, crisis plan)
- Forms for evaluating treatment consultations (e.g. did you and your treatment provider discuss what you needed?).

The app was developed in the period of 2013–2014 in the Mental Health Services of the Capital Region of Denmark in a process of co-creation between researchers, mental health providers and people with schizophrenia-spectrum disorders. Afterwards, the app was tested in a pilot study including 116 mental health professionals and 78 patients from three different mental health treatment sites: community of mental health, inpatient- and outpatient treatment sites. Based on the pilot study, the app was assessed most relevant in outpatient treatment sites [32]. The app has since in a development process with the Monsenso mHealth solution for mental health been optimized by incorporating the functionalities of preparing and evaluating treatment consultations from the app used in the pilot study, while adding options for daily self-evaluation and goal setting. This optimization was done based on the feedback received from the study participants in the pilot study. The app is in the trial driven by Monsenso ApS.

## Methods/Design

The aim and purpose of this trial is to investigate the effects of using the smartphone app for people with a diagnosis of schizophrenia, schizotypal or delusional disorders within outpatient treatment settings in a randomized design on selected outcomes. Our main hypothesis is that patients using the smartphone app in combination with receiving specialized early intervention treatment (which in this study is treatment as usual (TAU)), compared to patients only receiving TAU, will show greater improvements in patient activation, measured by the Consumer Health Activation Index – Mental Health version (CHAI-MH), 6 months after baseline (primary outcome). Our secondary hypothesis is that patients using the smartphone app in combination with receiving TAU, compared to patients only receiving TAU, will show greater improvements regarding self-perceived patient preparedness for SDM; self-efficacy; the therapeutic alliance; severity of symptoms; level of functioning; hope and optimism; satisfaction with treatment and; patient’s confidence in communicating preferences and concerns to their provider (secondary outcomes). Lastly, we will investigate whether there is a correlation between the effects of using the smartphone app and self-reported usefulness of the smartphone app and/or app usage (user sessions per day, screen views per day, screens per session, session duration and user retention) (explorative outcomes).

**Fig. 1:**
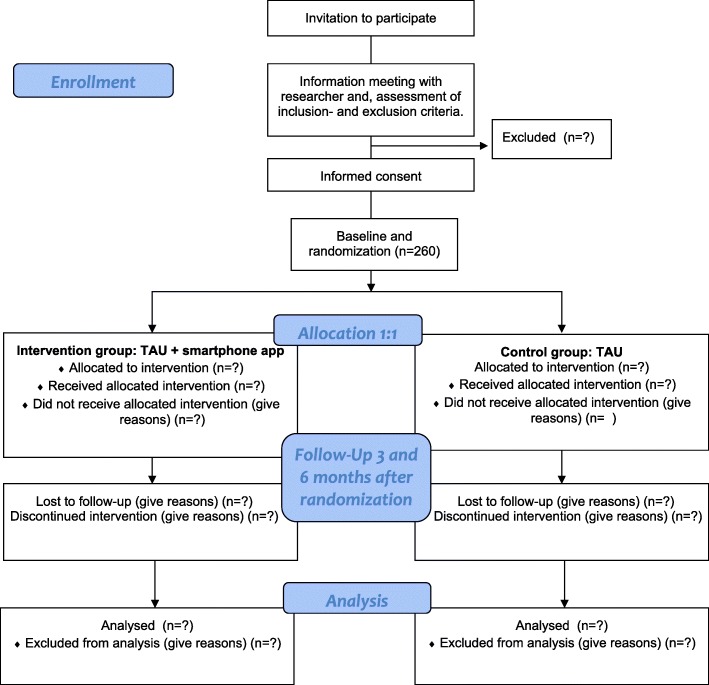
Flow-chart for The Momentum Trial

The Momentum trial is designed as a multi-centre, two-arm, parallel-group, observer-blinded block randomised controlled trial. Based on a power calculation a total of 260 participants, 130 in each arm will be recruited from five outpatient treatment sites called OPUS in the Capital Region of Denmark. Five sites were chosen based on their current number of patients and their yearly uptake of new patients. OPUS teams provide specialized early intervention (SEI) treatment to patients with a debuting diagnosis of schizophrenia or related psychotic disorders in the age group of 18–35 in Denmark. For this study, SEI treatment in OPUS will be considered as treatment as usual (TAU). The trial will compare two groups receiving TAU with one of the groups also using a smartphone-based aid to promote patient activation and support SDM (see Fig. 1).

### Description of study participants

Patients will be referred to the research team through their OPUS contact person. Patients will be eligible for the study if the patient meets the inclusion and exclusion criteria.

#### The inclusion criteria of the study are:

- Adults of both sexes aged 18+
- A diagnosis of schizophrenia, schizotypal or delusional disorder (ICD-10 codes: F20–F29)
- The patient has received treatment for a maximum of 18 months at the start of the intervention from one of five participating OPUS centres in the Mental Health Services of the Capital Region of Denmark

#### The exclusion criteria of the study are:

- Do not understand or speak Danish
- Unable to give written informed consent to participate in the trial at the described terms
- Are participating in other research studies involving OPUS treatment and an app
- Do not have daily access to a smartphone
- Are suffering of cognitive impairment or dementia (F. 70-F.79, F.00-F.03)

#### Criteria for discontinuing the intervention:

- If the patient stops his/her OPUS treatment or withdraws informed consent, they will be excluded from the intervention. Patients not receiving the full intervention will still be assessed at follow-up.

### Enrolment

The first contact with potential participants is done through the patient’s primary contact person in OPUS who has been informed about the Momentum trial. The patient will be provided with a written information sheet about the Momentum study by the contact person. The primary contact person will evaluate in collaboration with the patient whether the patient is fit to participate in the study or not. If the patient is interested, the patient will be invited to an information meeting with a researcher. Patients will be informed that they are welcome to bring relatives or friends to the meeting. The researcher will assess whether the patient meets the inclusion criteria and deliver verbal and written information about the study in an undisturbed office. Patients who wish to participate will be required to give verbal and written informed consent. 2 weeks of reflection time prior to consent will be offered. The baseline assessments will be conducted when the informed consent has been obtained, (see Table 1).

**Table 1:**
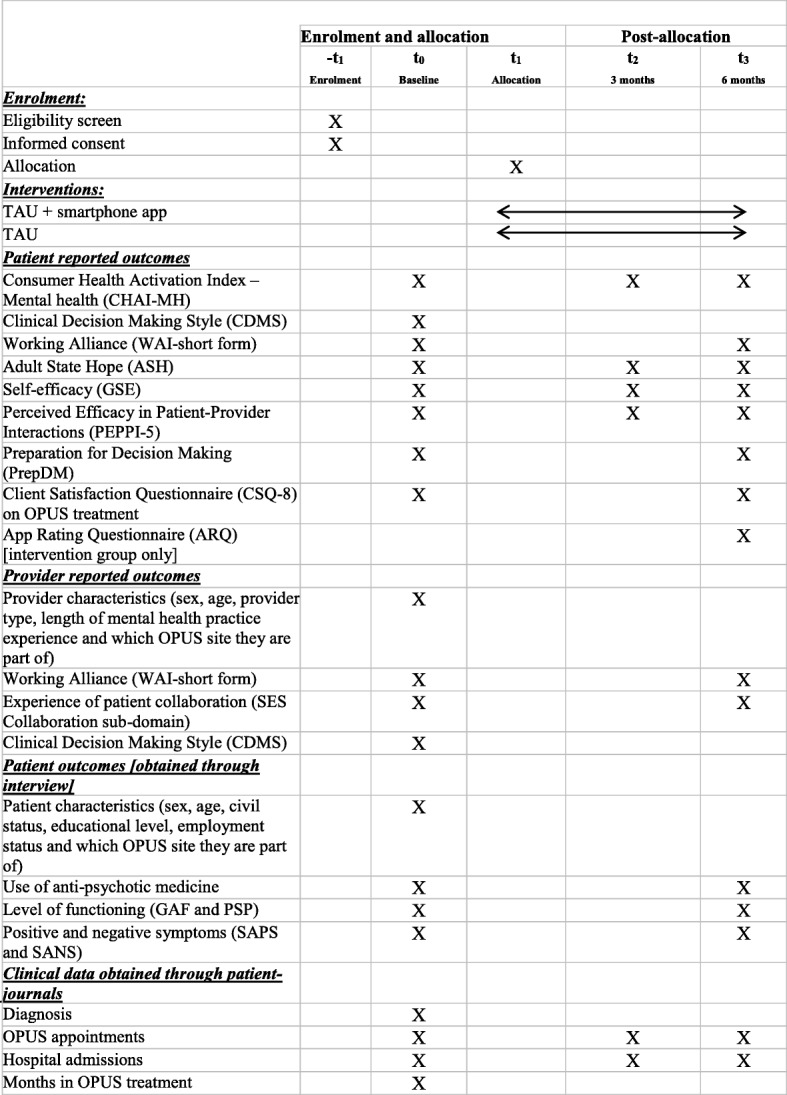
SPIRIT schedule of enrolment, interventions, and assessment

### Randomization and blinding

Randomisation to either the intervention group (TAU + smartphone app) or control group (only TAU) will be performed after baseline assessments. To achieve balance in the allocation of participants to treatment arms and to reduce risk of bias, block randomization of randomly determined block size will be used. To ensure adequate allocation concealment, the procedure will be centralised and computerised with concealed randomisation sequence facilitated by Odense Patient data Explorative Network (OPEN). Investigators will contact the randomisation unit, OPEN, with the necessary information to conduct the randomisation after baseline data have been collected.

Given the nature of the intervention, patients and healthcare providers cannot be kept blinded. Investigators responsible for data collection and data entry will be blinded as to patient allocation until all outcomes have been collected. Participants are given an identification number to ensure that the research team involved in data management are unaware of the treatment allocation. All patients are at each visit with the researcher thoroughly instructed not to mention anything about randomization allocation.

### Intervention

Patients allocated to the intervention group will receive TAU together with a manual-based introduction to the smartphone app with instructions on how to install and use the app. Once the app is installed, participants will be able to use the app until the end of the study. All participants are encouraged to use the app for 5 min per day to perform self-evaluation on parameters such as stress and sleep. Similarly, providers will be encouraged to use the web portal to check what input the patient has made in the app before a consultation (approximately once per week per patient). Both the control- and intervention group will receive TAU, which is provided by OPUS, a specialized assertive outpatient intervention for people age 18–35 years with a first episode psychosis. OPUS provides an integrated treatment approach by multidisciplinary staff (psychiatrists, psychologists, nurses, social workers etc.), including psychosocial interventions (e.g. psychoeducation and social skills training), for a period of 2 years. The treatment programme has been found to be effective at reducing negative- and psychotic symptoms [33]. Patients starting in OPUS are designated a healthcare provider, who has the responsibility as the patient’s primary contact person. All staff in OPUS may act as a primary contact person (except for the psychiatrist) and receives continually training and supervision in providing SEI treatment to people with a first episode psychosis. In collaboration with the patient, the treatment team assesses the most promising strategy to accommodate the patient’s needs and preferences. On average, primary contact persons in OPUS have 12–15 patients.

### Assessments

Communication between patient and provider (including SDM) may lead directly to improved health outcomes but more often communication affects health outcomes indirectly [18]. For instance, a SDM intervention could improve treatment satisfaction resolving in a promotion of health behaviour which lastly may end up improving different important health outcomes (e.g. reduction of symptoms). The conceptual framework by Shay and Lafata describes the link between SDM and affective-cognitive outcomes (e.g. alliance and satisfaction), behavioural outcomes (e.g. activation or engagement) and health outcomes (e.g. symptom reduction) [18]. Therefore, these areas will be measured during the intervention. Patient activation (behavioural outcome) was chosen as our primary outcome because of our intervention being primarily patient-centred. Prior to the data collection, we conducted a pilot study in OPUS with 8% of our proposed sample size (21 patients) to test the primary outcome (CHAI-MH). Furthermore, all questionnaires were tested with patients from OPUS to ensure that questions were understandable and unambiguous. The pilot test showed no ceiling effects or issues regarding the understanding of the questionnaires.

Participants will be assessed at three-time points: at baseline, after 3 months and after 6 months (end of intervention). Clinicians will be assessed at baseline and when each of their participating patients completes the 6 months intervention period. In case of non-respondents, participants will be contacted after 2 weeks with a reminder to complete the survey.

### Baseline parameters

For all patients enrolled, information on patient characteristics, diagnosis and duration in OPUS treatment will be collected. Data on diagnosis are used as an inclusion criterion while data on duration in OPUS treatment will be used to assess whether the smartphone app has a different effect based on the duration of receiving OPUS treatment. Background characteristics for the clinicians will also be collected. Since preferences in clinical decision making have been found to be related to patient involvement [34], the Clinical Decision Making Style questionnaire (CDMS) will be collected at baseline. The CDMS is a self-reported 21-item measure for the assessment of preferences in clinical decision making [16]. The scale consists of two subscales: 1) Participation in Decision Making (PD) and 2) Information (IN). The questionnaire is rated on a 5-point Likert scale and has been developed in two versions – one for patients and one for provider. The PD subscale has good internal consistency (Cronbach’s α = .85–.89), while the IN subscale has an acceptable internal consistency (Cronbach’s α = .75–.81). The PD and IN subscales do not correlate indicating convergent validity (i.e. preferences for participation in decision making are independent of preferences for information) [16]. In the present study, patients and their primary provider will be asked to complete the questionnaire to determine their level of agreement. The scale will only be used at baseline due to research indicating that the CDMS scoring is moderately to highly stable over 12 months [16].

### Primary outcome

The primary outcome of this trial is the difference in patient activation before participating in the trial and after 6 months participation. This will be assessed using the Consumer Health Activation Index for mental health (CHAI-MH) [35]. The 10-item instrument was developed based on 5 key domains: knowledge, self-efficacy, motivation and beliefs, actions, and internal locus of control. The questionnaire is rated on a 6-point Likert scale ranging from “Strongly disagree” to “Strongly agree”. Afterwards, the scores are transformed into a theoretical value from 0 to 100, with higher scores signifying a stronger involvement in the treatment and confidence in the ability to take care of one’s own health and health treatment. Higher CHAI scores have been associated with fewer depressive and anxiety symptoms. CHAI has been found valid and reliable while having good internal consistency (Cronbach’s α = .91) [35]. For this study the CHAI was modified to mental health. Hence, the mental health version of CHAI (CHAI-MH) has not yet been validated. The CHAI-MH was translated to Danish using a thorough forward/backwards translation process with three independent translators.

### Secondary outcomes

Based on the conceptual framework of Shay and Lafata [18], the study will investigate whether the smartphone app will improve the patient’s preparedness for SDM, self-efficacy, patient’s alliance with the clinician, severity of symptoms, level of functioning, hope, and treatment satisfaction. These outcomes will be obtained at baseline and after 6 months. In addition, the feeling of hope, self-efficacy and communication with one’s clinician will be obtained after 3 months.

The Preparation for Decision Making (PrepDM) scale is a self-reported 10-item instrument to measure the patient’s perception of how useful a decision aid or other decision support intervention is in preparing the respondent to communicate with their practitioner at a consultation focused on making health decisions [36]. The questionnaire is rated on a 5-point Likert scale from “Not at all” (1) to “A great deal” (5) with higher scores indicating a higher level of preparedness to communicate with one’s care provider regarding health decisions. The PrepDM has been validated with high internal consistency (Cronbach’s α = .92 to .96) [36]. The PrepDM scale will be translated to Danish for this study using the forward/backwards translation process. The General Self-Efficacy Scale (GSE) measures the patient’s belief that one’s actions are responsible for successful outcomes [37]. The questions consist of 10 items rated on a 4-point Likert scale ranging from “not at all true” (1) to “exactly true” (4) with higher scores indicating higher self-efficacy. The scale has been validated and found reliable in psychiatric care with the Danish version having good internal consistency (Cronbach’s α = .87) [38].

The Working Alliance Inventory-short (WAI) measures the therapeutic alliance between patients and clinicians within three domains; goals; tasks; and bond [39]. The questionnaire consists of 12 items rated on a 7-point Likert scale, with higher scores indicating better working alliance. The scale has been developed in two versions – one for patients and one for provider – and will in this study be filled out by patients and by the primary providers. Providers will fill out the instrument for each of their own patients participating in the study. The scale has good internal consistency (Cronbach’s α = .83–.98) and high correlation with the original WAI [40].

Perceived hope and optimism will be measured using the Adult State Hope Scale (ASH). The 6-item instrument is rated on a Likert-scale from 1 to 8, with higher scores indicating higher levels of hope [41]. The scale has been validated and shows good internal consistency (Cronbach’s α = .93). Psychometrically testing in various settings has shown the scale to be appropriate for self-report on hope and optimism in people with severe mental illnesses [42].

The Perceived Efficacy in Patient-Physician Interactions Questionnaire (PEPPI) is a self-reported 5-item instrument for the assessment of patient’s confidence in communicating with their physician [43]. The questionnaire is rated on a 10-point Likert scale, with higher scores indicating higher perceived efficacy in the interactions. The scale has been found to be valid and reliable in somatic care with excellent internal consistency (Cronbach’s α = .92) [43]. The scale will be translated to Danish for this study using the forward/backwards translation process.

The Client Satisfaction Questionnaire (CSQ) is a self-reported 8-item measure to assess a patient’s satisfaction with treatment. Each item is scored on a 4-point Likert scale, with higher scores indicating higher satisfaction. The CSQ has shown good psychometric properties with a high internal consistency (Cronbach’s α = .93) [44].

The Service Engagement Scale (SES) is a 14-item measurement for the provider to rate their patient’s engagement level on four subscales ‘availability’, ‘collaboration’, ‘help seeking’ and ‘treatment adherence’ [45]. Each item is scored on a 4-point Likert scale, with higher scores indicating poorer engagement. For this study, only the subscale ‘collaboration’ will be used, consisting of 3 questions. The SES has shown good psychometric properties and has previously been used in studies with patients with psychotic disorders [45].

To assess positive and negative symptoms, the Scale for the Assessment of Positive Symptoms (SAPS) and Scale for the Assessment of Negative Symptoms (SANS) will be used. The two scales consist of 3 dimensions: the psychotic, the disorganized, and the negative. Each dimension is scored between 0 and 5 with higher scores indicating higher symptom severity. Social functioning will be assessed using the Global Assessment of Function (GAF) and Personal and Social Performance Scale (PSP). GAF assesses a person’s psychosocial functioning (scores 1–100), with higher scores indicating a higher functioning level. GAF has been found highly generalizable and has been validated as a useful means of assessing global psychosocial health in people with psychotic symptoms [46, 47]. PSP assesses a person’s functioning level within four domains; socially useful activities; Personal and social relationships; Self-care; Disturbing and aggressive behaviors. PSP is scored 1–100, with higher scores indicating a higher functioning level. PSP has been found reliable and valid as a measure of social functioning in patients with schizophrenia [48].

Another aspect of interest is on whether the use of the smartphone app may affect adherence to treatment appointments, medication and/or hospital admissions. To measure this, data on patient adherence to OPUS appointments and number of hospitalizations will be collected from the patient journals while information on the usage of anti-psychotic medication will be collected through the patient interviews.

### Explorative outcomes

To explore the intervention group’s satisfaction of the app, the App Rating Questionnaire (ARQ) will be used in combination with objective data on app usage (user sessions per day, screen views per day, screens per session, session duration and session instances, user retention). The ARQ seeks to capture the end users perceptions of a software application [49]. The scale consists of 4 items scored on a 4-point Likert scale ranging from “Strongly agree” to “Disagree”.

### Data management

All the above-mentioned outcomes except for SAPS, GAF and PSP are either patient- or provider-reported outcome measures and will be collected through self-administered web-based questionnaires. Patients will complete the questionnaires on an iPad provided by the researcher. Questionnaires in paper format will be offered as an alternative. The completion of the questionnaires will be done in the presence of a researcher to clarify any uncertainties participants may have regarding the questionnaires. Questionnaires will be collected and managed using REDCap electronic data capture tools hosted at the The Mental Health Services of the Capital Region [50]. REDCap (Research Electronic Data Capture) is a secure, web-based application designed to support data capture for research studies. REDCap has a complete log and audit trail and complies with Danish legislation on processing personal data. Access to study data will only be available for data managers. Participants will receive an e-mail with a link to the questionnaires. Participants will have to login to a secure portal using a unique password, which is delivered at the patient’s meetings with the researcher. Interview-based data (such as SAPS, SANS, GAF and PSP) will be written directly into REDCap by the researcher. Information on diagnosis, hospital admissions, duration in OPUS treatment and adherence to treatment appointments will be collected retrospectively and retrieved from the patient journals.

### Power and sample size

The power calculation is based on the results of 6 RCT studies investigating patient activation using the Patient Activation Measure as a primary outcome [51–56], and by using the PS Power and Sample Size Calculations program version 3.1.2. This was done due to a lack of RCTs using the CHAI scale. While these studies use a different scale, they do measure the same domain: patient activation. We calculated our planned sample size based on the effect size on patient activation for these 6 studies. These studies had a mean effect size of 0,42, ranging from 0,28 – 0,65. Based on these studies, we expect to find an effect size between our intervention and control group of 0,42. To reach a power 80% and alpha of 0,05 we will need 90 participants in each group to be able to reject the null hypothesis that the population means of the two groups are equal. We plan on having 1 control per intervention subject. With an expectation of approximately 30% dropouts, we will include 130 participants in each group for a total of 260 participants. In addition, we performed sample size calculations for several secondary outcomes. None of these calculations exceeded 260 participants to detect a significant statistical difference for some of our secondary outcomes as well as our primary outcome.

### Statistical analysis

The analysis will be performed by using the IBM SPSS and SAS. Significance levels below 0,05 will be considered significant for the analysis of primary and secondary outcomes.

The data analysis will be conducted accordingly to the principle of intention-to-treat (i.e. patients will be included in the analysis according to group assignment even if they do not receive all of the planned intervention). In case of missing data, the operation of multiple imputations will be used to address the issue of missing values. To validate the complete case analyses, an analysis of the drop outs will be conducted.

With multiple assessment points, we will use mixed-effects linear regression for the primary analysis. The mixed-effects model is chosen to account for the dependency inherent in the data due to repeated measurements and to allow respondents with missing observations at different time points. If relevant, subsequent statistical tests will be performed. Cohens Kappa will be performed to determine the level of agreement between the patients and providers..

If any of our outcomes do not meet the assumptions for parametric statistical tests, data will either be transformed as appropriate prior to analysis or replaced by non-parametric statistical tests.

## Discussion

The Momentum trial is to our best knowledge the first randomized controlled trial to evaluate the effectiveness of a smartphone application to engage people with schizophrenia-spectrum disorders in their treatment consultations to support patient activation and SDM. The intervention will focus on supporting patients through the smartphone app to become more active in their care (primary outcome). In addition, the intervention will also connect patients together with their providers (through a web portal) to gain a common starting point before treatment consultations. We expect that the combination of patients becoming more active in their care together with having their data shared with the provider will improve the patient’s preparedness for SDM, the therapeutic alliance, the efficacy of therapeutic interactions, hope, self-efficacy, severity of symptoms and level of functioning (secondary outcomes).

A major strength of the study is the comprehensive pilot study prior to the trial. While the trial is primarily focusing on quantitative assessments, the pilot study was mainly qualitative with a focus on identifying needs and preferences in the target group in relation to a smartphone app to support SDM. Another strength of the study is the focus and assessment on both patient and provider. Research indicates that addressing data from patients and providers together may yield more powerful effects than only focusing on one of the two [8]. Information from both perspectives will also provide valuable information on how patients and providers view their alliance. In addition, providers will be asked to rate the patient’s engagement level to compare this with the patient’s own perceived level of engagement. Finally, we have a solid sample size calculation to perform a data analysis with good strength regarding our primary outcome and several secondary outcomes.

The methodical limitations in this study may be that since providers are not blinded and potentially are going to have participating patients from the control- and the intervention group, a contamination bias could occur. Providers may gain experience in the concepts of SDM from working with patients in the intervention group which they then could apply (intentionally or unintentionally) to their patients in the control group. The study is also at risk of a recruitment bias if providers mainly recruit patients who are rather stable and therefore not completely representable of OPUS patients. To address this, we will throughout the recruitment period encourage providers to inform all of their patients about the study regardless of the patient’s level of functioning. Based on prior studies using mobile applications, we estimate a drop out level of 30%. To address the risk of patients not using the app consistently, the user will have the option within the app to setup daily reminders to use the app. A drop out analysis will be conducted to identify potential characteristics of those dropping out or who stop using the app.

A limitation for this study is the lack of qualitative data. Qualitative interviews performed after the intervention to explore the participants’ experiences of the smartphone app in relation to SDM is being considered. Observational measures on the interactions between patient and provider to observe potential changes in the decision-making process and on how the app is used in the consultations would be meaningful. If feasible, interviews and observational measures will be performed after the end of the intervention. Although patients and providers are encouraged to include the smartphone app in the consultations when relevant, it is unknown to what extent this will happen. To address this, patients in the intervention group will be asked at the end of the intervention approximately how often they included the app in the consultation and whether it was the patient or the provider who took the initiative to include the app. Although OPUS is a well-established treatment site, OPUS was chosen as our study site due to providing a high feasibility for conducting research studies and due to our pilot study indicating that younger adults with schizophrenia-spectrum disorders indicate that an easy accessible solution, such as the smartphone app presented in this trial is a relevant tool to better prepare for treatment consultations and to take a more active role in the treatment [32]. Patients receiving OPUS treatment meet with several staff members. Still, we chose only to focus on the primary provider due to the fact that a patient in OPUS meets their primary provider significantly more often than any of the other staff members (e.g. the psychiatrist).

If the Momentum trial confirms the hypothesis that providing people with schizophrenia-spectrum disorders with the presented system will increase their level of activation in the treatment and support SDM, the study will provide evidence on the effects of using such tools to support people with severe mental illness in engaging in their own healthcare management. In addition, the study may provide evidence to the idea that linking client and practitioners in digital solutions can have advantages in facilitating SDM in mental health. Overall, the study will provide more evidence to the further developing and implementation of electronic tools to support SDM in mental health care. Future enhancements will be to integrate the smartphone app with the existing IT systems and to investigate its efficacy long term (> 1 year) and in other mental healthcare settings.

## Trial status

Inclusion of participants started in January 2019 and is expected to end April 2020.

## Abbreviations

ARQ: App Rating Questionnaire
ASH: Adult State Hope Scale
CDMS: Clinical Decision Making Style
CHAI-MH: Consumer Health Activation Index – Mental Health version
CSQ-8: Client Satisfaction Questionnaire-8 items
GAF: Global Assessment of Function
GSE: General Self-Efficacy Scale
ICD-10: International Statistical Classification of Diseases and related Health Problems, 10th revision
PEPPI-5: Perceived Efficacy in Patient-Provider Interactions-5 items
PrepDM: Preparation for Decision Making
PSP: Personal and Social Performance Scale
RCT: Randomized Controlled Trial
SAPS/SANS: Scale for the Assessment of Positive Symptoms/Scale for the Assessment of Negative Symptoms
SDM: Shared Decision Making
SEI: Specialized Early Intervention
SES: Service Engagement Scale
TAU: Treatment as usual
WAI-S: Working Alliance Inventory-Short form
